# The Evolution of a Microstructure during Tempering and Its Influence on the Mechanical Properties of AerMet 100 Steel

**DOI:** 10.3390/ma16216907

**Published:** 2023-10-27

**Authors:** Hongli Wang, Jian Zhang, Jingtao Huang, Chengchuan Wu, Xianguang Zhang, Zhonghong Lai, Yong Liu, Jingchuan Zhu

**Affiliations:** 1 School of Materials Science and Engineering, Harbin Institute of Technology, Harbin 150006, China20b909032@stu.hit.edu.cn (J.H.); 2 Chengdu Advanced Metal Materials Industrial Technology Institute Co., Ltd., Chengdu 610300, China; 3 State Key Laboratory of Metal Material for Marine Equipment and Application, Anshan 114009, China; 4 School of Metallurgical and Ecological Engineering, University of Science and Technology Beijing, Beijing 100083, China; xgzhang@ustb.edu.cn; 5 Center for Analysis, Measurement and Computing, Harbin Institute of Technology, Harbin 150001, China

**Keywords:** carbide, reverted austenite, AerMet 100 steel, K*_IC_*, strength

## Abstract

In order to provide guidance for furthering the balance of strength and toughness of AerMet 100 steel through tempering treatment, the effects of the tempering time on microstructure and mechanical properties are investigated. The microstructure evolution, especially M_2_C precipitates and austenite in AerMet 100 tempered at 482 °C for 1~20 h, was characterized, and its influences on the mechanical properties were studied. The tensile strength decreases gradually, the yield strength increases first and then decreases, and the fracture toughness K*_IC_* increases gradually with an increasing tempering time. The strength and toughness matching of AerMet 100 steel is achieved by tempering at 482 °C for 5~7 h. Without considering the martensitic size effect, the influence of the dislocation density on the tensile strength is more significant during tempering at 482 °C. The precipitation strengthening mechanism plays a dominant role in the yield strength when tempering for 5 h or less, and the combined influence of carbide coarsening and a sharp decrease in the dislocation density resulted in a significant decrease in tensile strength when tempering for 8 h or more. The fracture toughness K*_IC_* is primarily influenced by the reverted austenite, so that K*_IC_* increases gradually with the prolongation of the tempering time. However, a significant decrease in the dislocation density resulting from long-term tempering has a certain impact on K*_IC_*, giving rise to a decrease in the rising amplitude in K*_IC_* after tempering for 8 h or more.

## 1. Introduction

High Co-Ni secondary hardening steels are the main metal materials used for aeronautical structures, and AerMet 100 steel is one of the most typically used steels for landing gear applications. The strength and toughness of materials used for landing gear applications greatly affect the lifetime and security of an aircraft, so the balance of strength and toughness of the material is of vital importance. High Co-Ni secondary hardening steel is a group of metal materials with an excellent balance between the strength and toughness [1,2,3,4,5,6], especially AerMet 100 steel. Previous studies have indicated that the tempering temperature had a significant impact on the strength and toughness. The strength decreases and the K*_IC_* increases with the increase in the tempering temperature around 482 ± 10 °C, which is mainly attributed to the growth of M_2_C carbide and the reverted austenite film [3]. The strength and toughness of AerMet 100 can achieve a relatively nice balance, meeting the landing gear application requirements of advanced aircrafts.

The improved strength of AerMet 100 steel is mainly induced by the precipitation of M_2_C carbides during the tempering progress [7,8,9]. The peak strength of the secondary hardening for AerMet 100 steel could be obtained by tempering around 482 °C, which gives rise to the full precipitation of M_2_C carbides [10,11]. The M_2_C carbides in high Co-Ni secondary hardening steels are needle-shaped and coherent with a martensitic matrix at the stage of peak secondary hardening [10,11,12,13,14], and it has been proven that the M_2_C carbides have a hexagonal structure [15,16,17,18,19,20]. Recent research results indicate that [7] M_2_C carbides with a hexagonal structure firstly nucleated and then grew slowly in the case of AerMet 100 steel during tempering at 482 °C, and this process was dominated by needle-shaped precipitates when tempering for 7 h or less. The hexagonal M_2_C transformed into an orthogonal structure with atomic-scale lamellar-shaped precipitates, which tend to be coarsened with the increase in tempering time, leading to a decrease in the total strength of the steel when tempering at 482 °C for 7 h or more.

The toughening behavior of high Co-Ni secondary hardening steels was due to the reversed austenite at the lath and block boundaries of martensite, as well as a decrease in the dislocation density during the tempering progress, owing to the fact that the austenite layer could prompt the advancing crack to divert, branch, and become blunt [17]. Previous research, using simulation, has indicated that the thickness of the austenite layer had a significant effect on the fracture toughness of the steels with martensite as matrix, but the angles between the austenite layer and the initial crack propagation direction had little effect on K*_IC_* [18]. At present, one explanation for the mechanism of austenite reverse transformation during the tempering process of high Co-Ni ultra-high strength steel is that [19] the formation of reverted austenite during tempering is dependent on a redistribution of substitutional atoms such as Ni, Co, and Cr; that is, the occurrence of the austenite’s reverse behavior is based on the significant difference in the diffusion and migration rates of these atoms in martensite and the retained austenite during the tempering process. Ni and Cr enrichment and Co depletion occurs in the interface of the neighboring martensite during the process of tempering. When the stabilizing element of austenite mainly refers to the Ni concentration reaching the level of austenite transformation, the transformation from martensite into austenite will occur. The chemical composition of the reversed austenite with Ni-rich regions is fundamentally different from that of the residual austenite, resulting in a higher stability against phase transformation into martensite during plastic deformation, and thus, the reversed austenite has a decisive influence on the toughness of the material.

With the development of the aeronautical industry, there is a higher demand for the lifetime and safety of landing gear, so progressive research on the balance between strength and toughness and the mutual influence of them is also necessary for its application. The microstructure evolution of high Co-Ni secondary hardening ultra-high strength steel during tempering at even 482 °C is complex, and its influence on mechanical properties also needs further exploration. A tempering temperature of 482 °C is adopted to study the microstructure evolution and its influence on the mechanical properties of AerMet 100 steel with various tempering times, providing guidance for further balancing of strength and toughness.

## 2. Materials and Methods

The chemical composition of the AerMet 100 steel used in this work is shown in Table 1. The samples taken from a forged steel bar were austenitized at 885 °C for 1 h and quenched in oil to room temperature, then immediately transferred to a cryogenic bath at −73 °C for 1 h and tempered at 482 °C; the tempering times were 1, 3, 5, 7, 8, 10, and 20 h, respectively. In order to minimize experimental variables and reduce the difference in martensite size of samples with different tempering times, all samples used in this study are taken from the half radius of the same round bar of steel.

**Figure 1:**
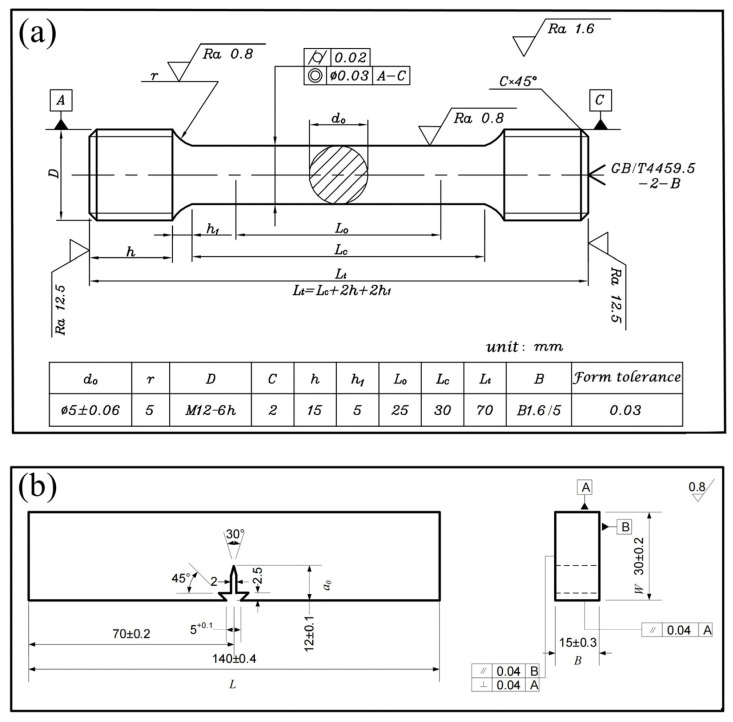
Specimen dimensions for mechanical measurements: (**a**) uniaxial tensile specimen; (**b**) fracture toughness specimen.

The uniaxial tensile tests in this study were conducted using a CMT 5105 electron-mechanical universal testing machine at room temperature according to the national standard GBT228.1-2010 [21], with a loading rate of 0.5 mm/min. The tensile sample sizes were a diameter of 5 mm and a gauge length of 25 mm, as shown in Figure 1a. Two uniaxial tensile specimens were tested for each tempering condition. Two fracture toughness specimens, as shown in Figure 1b, were prepared for each tempering process. Both the first fatigue cracking of the notches and the final loading to fracture were carried out using the MTS 370.10 voltage and hydraulic servo fatigue machine, using the standard ASTM E399-09 [22]. All fracture toughness specimens were taken in L-C orientation, and the final crack limit of fatigue pre-crack was 15 mm. Another standard fracture toughness specimen, tempered at 482 °C for 5 h, was terminated when the maximum load (P_max_) that the sample could withstand appeared during the standard fracture toughness (K*_IC_*) experiment. 

The austenite fraction and dislocation density in AerMet 100 steel were measured via XRD, and the samples were subjected to Co-Ka radiation with a scanning speed of 1°/min. The M_2_C precipitates and austenite of the steel were characterized via high-resolution spherical aberration-corrected transmission electron microscopy (TEM, FEI Titan-ETEM) at 300 kV. Thin foils for TEM observations were prepared through twin-jet electron polishing in a 10% perchloric acid and 90% ethanol solution at a temperature of −30 °C and a voltage of 20 V. The morphology of the crack front and microstructure near the crack in the specimen tempered at 482 °C for 5 h, which the K*_IC_* experiment established, were observed via electron back-scattered diffraction (EBSD). The sample for EBSD was prepared through standard mechanical polishing and then vibration polishing to remove the surface stress, using a PRESI SA 230 VAC vibration polisher. EBSD scans were carried out using a JEOL JSM-7900F field-emission scanning electron microscope operated at 20 kV with scanning steps of 0.1 µm. The channel 5 and Aztec v2.1 software provided by Oxford Inc. were used for EBSD data analysis. 

The precipitations of M_2_C carbides were studied via kinetic calculations using the software package TC-PRISMA v2021b^TM^. Meanwhile, the thermodynamic database TCFE9 and the mobility database MOBFE4 were used.

## 3. Results

### 3.1. Microstructure Analysis

**Figure 2:**
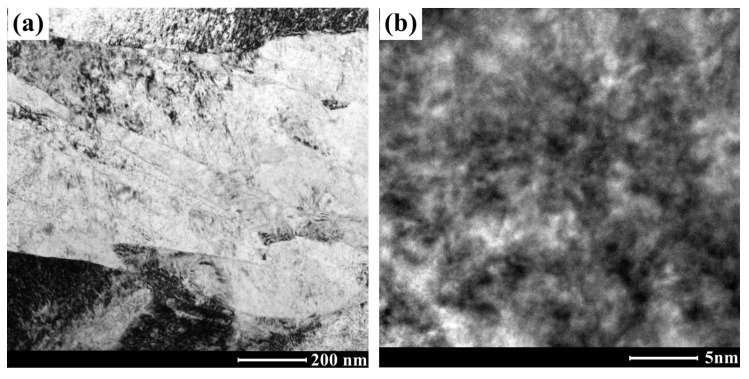
Microstructure of AerMet 100 steel before tempering observed via TEM: (**a**) bright field image; (**b**) HRTEM image taken along the [001]_M_ zone axis.

The typical microstructure of the AerMet 100 steel before tempering was investigated via TEM, as shown in Figure 2. It could be seen from the TEM results that the microstructure before tempering was lath martensite, with a lath width of around 100~200 nm, as shown in Figure 2a. In the martensitic lath taken along the [001]_M_ zone axis, there were high density dislocations but no precipitates, as shown in Figure 2b.

**Figure 3:**
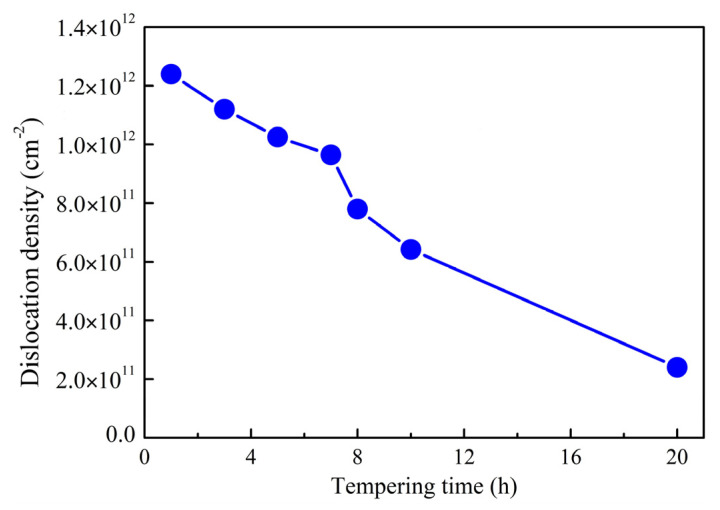
Dislocation density of microstructure in samples when tempering for various hours.

As shown in Figure 3, the AerMet 100 steel maintained a high dislocation density during tempering at 482 °C. The dislocation density is no less than 1.0 × 10^12^ cm^−2^ when the tempering time is within 5 h, and significantly decreases with the prolongation of the tempering time when tempering occurs for more than 7 h.

#### 3.1.1. Precipitation of M_2_C Carbides

**Figure 4:**
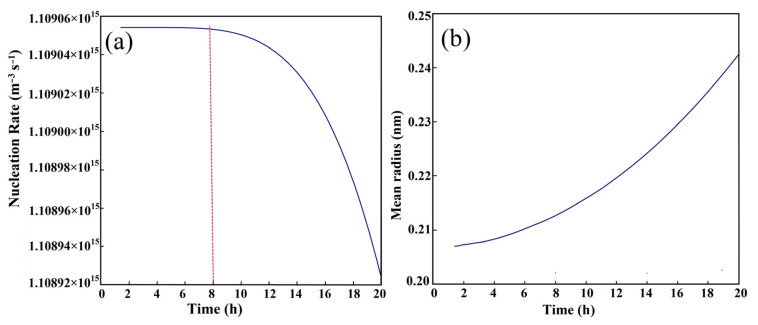
The evolution of M_2_C carbide with hexagonal structure nucleated at dislocation as a function of tempering time at 482 °C for AerMet 100 steel predicted via TC-PRISMA. (**a**) Nucleation rate; (**b**) mean radius.

The evolution of M_2_C carbides with a hexagonal structure, nucleated at dislocation and predicted via TC-PRISMA, is shown in Figure 4. The incubation period for the nucleation of hexagonal M_2_C carbides at dislocation was approximately 1 h. The hexagonal M_2_C nucleated at the maximum rate after the incubation period, moreover, still had good nucleation kinetic conditions when tempering at 482 °C for more than 8 h. It was indicated that M_2_C has sufficient nucleation kinetics conditions to form a large number of crystal embryos or tiny M_2_C carbides with a stable crystal structure, and the mean radius of the tiny carbides is about 0.2 nm in the early stage of tempering at 482 °C (for more than 1 h), as displayed in Figure 4b.

The mean radius of the M_2_C carbides in AerMet 100 steel was calculated based on the constant volume law, as shown in Equation (1) [5].

(1) $$\frac{4}{3}\pi {\bar{R}}^{3}=\pi r^{2}l$$

where $\bar{R}$ is the mean (equivalent) radius of the M_2_C carbides, and *r* and *l* are the actual radius and length of the needle-shaped M_2_C carbides, respectively.

**Figure 5:**
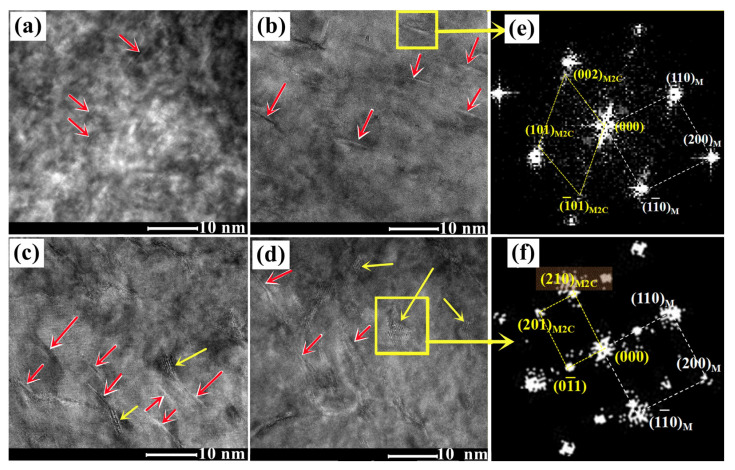
Typical HRTEM micrographs of M_2_C carbide in AerMet 100 steel tempered at 482 °C for (**a**) 1 h; (**b**) 3 h; (**c**) 5 h [7]; (**d**) 7 h [7]. (**e**,**f**) represent the FFT patterns of M_2_C in (**b**,**d**), respectively. The needle-shaped precipitates are marked by the red arrows, while atomic-scale lamellar-shaped precipitates are marked by yellow arrows.

The typical HRTEM images taken along the [001]_M_ zone axis of the samples subjected to tempering conditions at 482 °C for 1~7 h are shown in Figure 5. The needle-shaped precipitates with black–white contrast in the sample after a tempering process for 1 h are observed, as marked by the red arrows in Figure 5a, and the radius and length of the needle-shaped precipitates are about 0.2 nm and 1~2 nm, respectively, which is almost consistent with the dynamic calculation results in Figure 4b. The corresponding fast Fourier transform (FFT) pattern of these precipitates could not be obtained. Firstly, the carbides are too small in size, and it may also be due to the fact that they are in the element-rich zone instead of forming the carbides with a stable crystal structure of M_2_C, which is similar to the results reported for M54 steel [20]. The size of the carbide precipitates significantly increases, with a length of about 3~5 nm when tempering for 3 h, as shown in Figure 5b. The carbides have a hexagonal structure under the current tempering condition, according to the corresponding FFT diffraction (Figure 5e). The atomic-scale lamellar-shaped precipitates (marked by yellow arrows) could be observed when tempering for 5 and 7 h, as shown in Figure 5c,d [7]. The corresponding FFT diffraction (Figure 5f) indicated that they can be identified as orthorhombic-structure M_2_C carbides. The size of the lamellar-shaped carbides is increased, and the significant precipitation occurred with the prolongation of the tempering time. Meanwhile, the orthorhombic M_2_C is easily coarsened against the tempering time [7]. 

**Figure 6:**
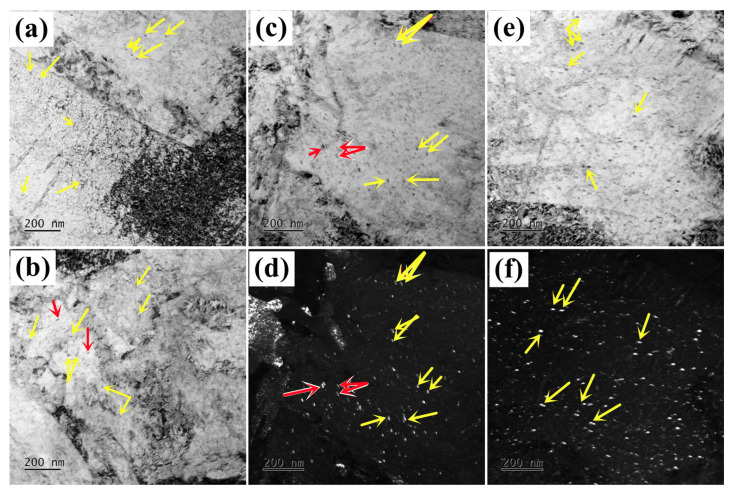
Typical TEM micrographs of M_2_C carbide in AerMet 100 steels tempered at 482 °C for (**a**,**b**) 8 h; (**c**,**d**) 10 h; (**e**,**f**) 20 h. The needle-shaped precipitates are marked by the red arrows, while atomic-scale lamellar-shaped precipitates are marked by yellow arrows.

Figure 6 displays typical TEM micrographs of M_2_C carbide in AerMet 100 steel tempered at 482 °C for more than 7 h. Most of the needle-shaped carbides with a hexagonal structure are transformed into orthogonal structures with small aspect ratios when tempering for 8 h, as marked by the yellow arrows shown in Figure 6a,b. The bright-field and corresponding dark-field TEM images indicated that the high-density orthorhombic carbides are dispersed, and the size of carbides significantly increases with the prolongation of the tempering time when tempering for 10 h or more, as shown in Figure 6c–f. 

The hexagonal M_2_C nucleated at the maximum rate after the incubation period when tempering at 482 °C for approximately 1 h, resulting in a high density of M_2_C precipitates. The M_2_C carbides with a hexagonal structure maintain a tiny size, indicating that the hexagonal carbides grow slowly when tempered for less than 5 h. The volume fraction of M_2_C carbides, including hexagonal and orthogonal structures, increased obviously, which is attributed to the transformation of the hexagonal structure into an orthogonal structure that is relatively easy to coarsen when tempered for 5 h or longer. The increase in size rather than quantity of carbides (as shown in Figure 6) indicates that the evolution of carbides mainly involves coarsening rather than nucleation when tempered for more than 7 h. This is attributed to a significant decrease in the dislocation density caused by the tempering and the occupation of nucleation sites (dislocations) by already nucleated M_2_C.

#### 3.1.2. The Evolution of the Austenite

**Figure 7:**
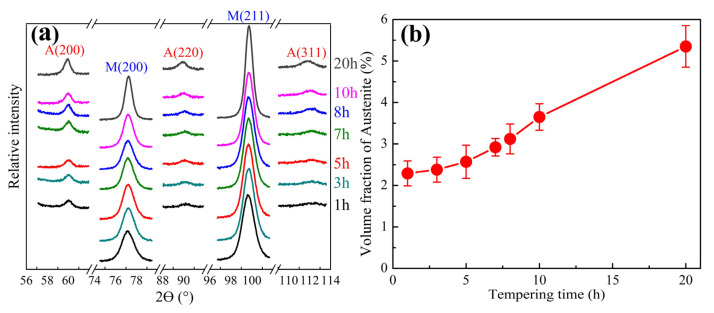
The volume of austenite as function of tempering time. (**a**) Volume fractions of austenite measured via XRD; (**b**) corresponding diffraction pattern.

The evolution of the austenite during tempering at 482 °C is shown in Figure 7. Overall, the volume fraction of the austenite increases approximately linearly with the prolongation of tempering time. The fraction of austenite in the tempered samples was significantly influenced by the tempering time; it increased from approximately 2.3 to 2.6% as the tempering time extended from 1 h to 5 h. There were indications that the growth of the austenite exhibits a relatively slow rate when tempered for 5 h or less. The austenite volume fraction rapidly increased from about 2.9 to 5.4% as the tempering time extended from 7 h to 20 h, which indicates that the growth rate of austenite increases when tempering for more than 5 h. This may be related to the precipitation and growth of M_2_C at different tempering time stages. 

**Figure 8:**
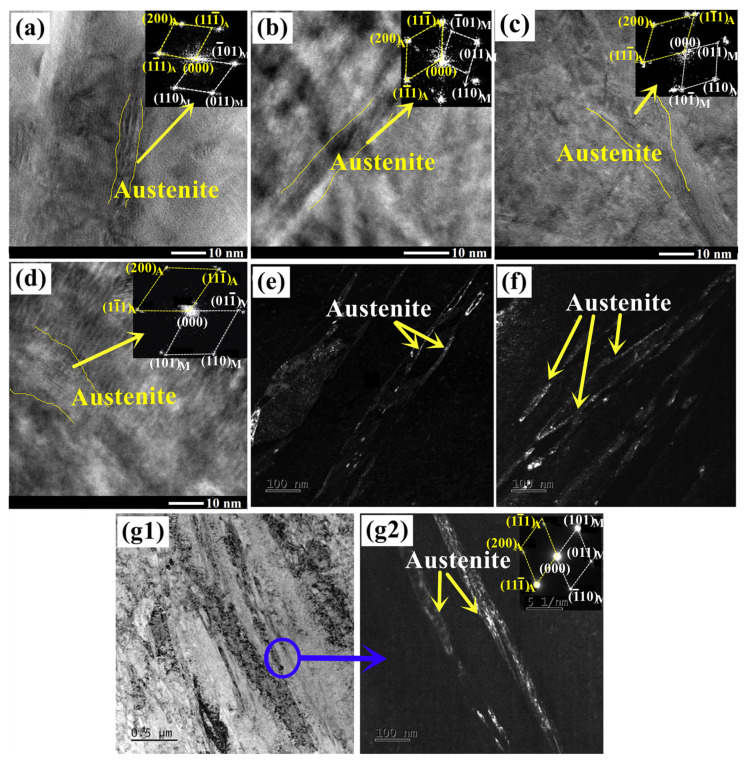
The TEM analysis of the austenite in AerMet 100 steel tempering for (**a**) 1 h, (**b**) 3 h, (**c**) 5 h, (**d**) 7 h, (**e**) 8 h, (**f**) 10 h, and (**g1**,**g2**) 20 h.

The typical morphology of the austenite in AerMet 100 steel tempered at 482 °C for varying times, observed via TEM, are shown in Figure 8. It could be seen that the tempered austenite presents in the form of strips or films. The width of the austenite is about 5 nm when tempered for 1 h, 6~8 nm for 3 h, 8~12 nm for 5 h, and approximately 15 nm for 7 h, as shown in Figure 8a–d. Figure 8e,f are the dark-field images of austenite in samples tempered for 8 and 10 h, respectively. Figure 8(g1) presents the bright-field images of austenite in the structure, and Figure 8(g2) shows the dark-field images of locally enlarged images in the blue circle of Figure 8(g1). Reversed austenite was observed at the martensitic lath boundaries and block or sub-block boundaries, which is consistent with most of the austenite being reversed from martensite in dual-phase steels [23,24,25]. It is discovered that the width of the austenite rapidly increases when tempering for 8 h or more. The width of the austenite could reach about 50 nm when tempered for 20 h. 

The evolution of the austenite width with an increasing tempering time is consistent with the volume fraction of austenite measured via XRD. According to previous research results [19,26], when investigating the mechanism of austenite reverse transformation during the tempering process of high Co-Ni secondary hardening ultra-high strength steel, it could be inferred that the reverse transformation of austenite depends on the diffusion and migration of austenite-stabilizing elements such as Ni and C in the matrix during the tempering process. The nucleation and precipitation of carbide occur simultaneously with austenite undergoing reverse transformation during the tempering process. From the results in Section 3.1.1, it can be seen that the nucleation rate of M_2_C carbides achieve their peak value, and the C atoms in the matrix mainly diffuse and migrate to form carbides, resulting in a lower austenite reversal rate when tempered for 5 h or less. However, the evolution of carbides gradually converts from nucleation- and growth-dominated to coarsening-dominated when the tempering time exceeds 7 h. In this case, the diffusion of C atoms in the matrix can also be used to support the reverse transformation of austenite, resulting in a relatively fast growth rate of austenite in this study. 

### 3.2. Mechanical Properties

**Figure 9:**
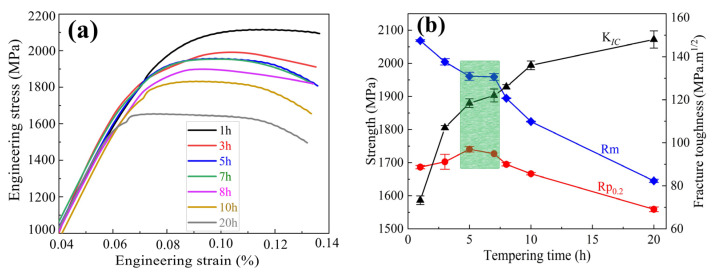
The mechanical properties of AerMet 100 steel results as a function of aging treatment: (**a**) Stress–strain curves of the tensile test; (**b**) Room-temperature tensile properties and fracture properties. Rm represents tensile strength, Rp_0.2_ represents yield strength, K*_IC_* represents fracture toughness.

The room-temperature tensile properties and fracture properties (K*_IC_*) results for samples tempered at 482 °C for various times are shown in Figure 9a, and the representative strain versus strain plots are measured in Figure 9b. The results indicate that the tensile strength decreased gradually as the tempering time prolonged. The yield strength increased and reached approximate peak values of 1732~1749 MPa and tensile strength of 1949~1973 MPa when the tempering time reached 5 h; after this, the yield strength decreased as the tempering continued. The fracture toughness K*_IC_* increased gradually when the tempering time increased. The K*_IC_* was in a range of 116.5~120.5 MPa m^1/2^ when tempering for 5 h. When tempering for 7 h, the tensile strength reached a range of 1975~1989 MPa, the yield strength amounted to 1727 MPa, and K*_IC_* corresponded to 119~122 MPa m^1/2^. In the present investigation, the AerMet 100 steel achieves excellent strength and fracture toughness matching when tempering at 482 °C for 5 to 7 h.

## 4. Discussion

Based on previous studies [7], there is a strong correlation between yield strength and M_2_C carbides in this study, as the strengthening effect of martensite size could be ignored. The yield strength Rp_0.2_ increased with the prolongation of the tempering time when tempered for 5 h or less and reached a peak at 5 h, which was due to the gradual nucleation and precipitation of needle-shaped nano-sized M_2_C carbides with a hexagonal structure. The hexagonal M_2_C carbide gradually transforms into an orthogonal structure that tends to coarsen as the tempering increases to more than 5 h. In fact, the carbides coarsened severely when tempered at 482 °C for 8 h or longer, so the yield strength decreased with the prolongation of tempering time. 

The yield strength of a material is the stress value at the beginning of plastic deformation, while the tensile strength represents its ultimate bearing capacity. During the tempering process, the tensile strength increases due to the precipitation of carbides and decreases with the decrease in dislocation density, as shown in Figure 3. Without considering the martensitic size effect, the dislocation strengthening plays a dominant role in the tensile strength when tempering for 5 h or less, resulting in the Rm decreasing with the prolongation of the tempering time. The combined influence of carbide coarsening and a sharp decrease in the dislocation density resulted in a significant decrease in the tensile strength when tempering for 8 h or more.

From the perspective of macroscopic fracture mechanics, the fracture toughness K*_IC_* is related to strength according to the derivation formula [3]:

(2) $$K_{IC}=\sqrt{\frac{8\times 10^{-4}[\frac{\left(E\delta_{u}-Rp_{0.2})(Rp_{0.2}+2Rm\right)}{3}+\frac{Rp_{0.2}^{2}}{2}}{1-\upsilon^{2}}}$$

where *E* is Young’s modulus of material, δ*_u_* represents the uniform elongation of the material, and υ is Poisson’s ratio. From Equation (2), it can be seen that K*_IC_* has a positive correlation with the yield strength *Rp*_0.2_ and tensile strength Rm; that is, a significant decrease in strength will lead to a decrease in K*_IC_* under other unchanged conditions. Therefore, the rising amplification of K*_IC_* decreases with the increase in the tempering time when tempering for more than 8 h.

**Figure 10:**
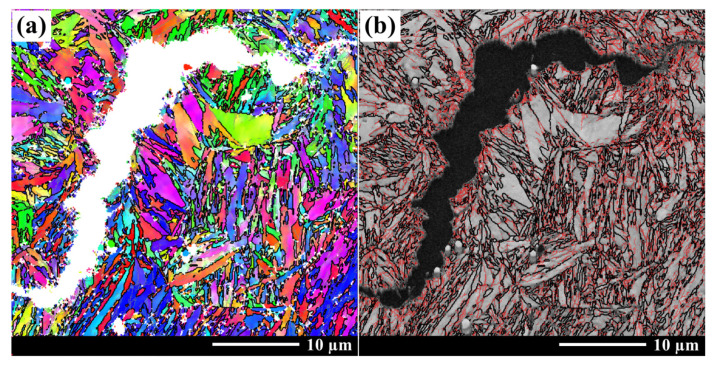
EBSD maps of the crack propagation. (**a**) IPF + GB, (**b**) BC + GB; grain boundaries of 2~15° are represented by red lines, and those greater than 15° are represented by bold black lines.

From the perspective of crack propagation and microstructure, the fracture toughness K*_IC_* is related to the reversed austenite and martensitic lath. The martensitic crystallographic information at the crack tip of the terminated K*_IC_* sample is observed using EBSD, as shown in Figure 10. The crack propagated along the martensitic laths and block boundaries, and this would prompt the advancing crack to divert and branch at the place where the small martensitic laths and block are placed, resulting in a large misorientation difference [26]. For high Co-Ni ultra-high strength steel, the high stability and plasticity of continuously reversed austenite films formed primarily at the martensitic lath or block boundaries during the tempering process could further promote the direction of crack propagation, leading to diverting, branching, and passivation, which could effectively reduce the stress concentration at the crack front and result in an increase in the fracture toughness [3,27]. Therefore, a small increase in reverse austenite can significantly improve the fracture toughness of the steel when tempered for 7 h or less. It could also be seen that crack propagation needs to cut through the martensite block due to the large martensite size. There was a significant reduction in the dislocation density in the martensite when tempering for 8 h or more, resulting in a reduced hindrance to crack propagation when cracks cut through the martensite, making crack propagation easier. As a consequence, the rising amplitude of K*_IC_* decreases with the prolongation of the tempering time when tempering for 8 h or more.

Above all, the tempering time at 482 °C has a significant impact on the strength and toughness of AerMet 100 steel. Without considering the martensitic size effect, the dislocation strengthening plays a dominant role in the tensile strength, resulting in Rm decreasing with the prolongation of the tempering time. The precipitation strengthening plays a dominant role in the yield strength as tempering for 5 h or less. The combined influence of carbide coarsening and a sharp decrease in the dislocation density resulted in a significant decrease in the tensile strength when tempering for 8 h or more. The fracture toughness K*_IC_* of AerMet 100 steel primarily depended on the reverted austenite when tempering for less than 8 h. The rising amplitude of K*_IC_* decreases as the tempering time increases when tempering for 8 h or more, due to a significant reduction in the dislocation density of the martensite.

## 5. Conclusions

The strength and toughness were measured, and the microstructure evolution of AerMet 100 steel tempered at 482 °C was studied via XRD and HRTEM. Meanwhile, the crack propagation in the K*_IC_* sample was analyzed using EBSD, and this leads to the following conclusions:

1. The tensile strength Rm decreases gradually, the yield strength Rp_0.2_ first increases and then decreases, and the fracture toughness K*_IC_* increases gradually with increasing tempering time at 482 °C from 1 h to 20 h.
2. The gradual nucleation and precipitation of the needle-shaped nano-sized M_2_C carbides with a hexagonal structure results in the increase in the yield strength Rp_0.2_ with the prolongation of the tempering time when tempered for 5 h or less. The hexagonal M_2_C carbides gradually transformed into an orthogonal structure that tended to coarsen as the tempering increased to more than 5 h. Significantly coarsened behavior can be observed when tempering at 482 °C for 8 h or longer, leading to the yield strength decreasing with the prolongation of the tempering time.
3. The fraction of reverted austenite increased with the prolongation of the tempering time, resulting in an increase in the fracture toughness K*_IC_*. The significant decrease in the dislocation density reduced the rising amplitude of K*_IC_* when tempering for 8 h or more.
4. The strength and toughness balancing of AerMet 100 steel is achieved through tempering at 482 °C for 5~7 h. A yield strength of 1727~1749 MPa, tensile strength of 1949~1989 MPa, and K*_IC_* value between 116.5 and 122 MPa·m^1/2^ are obtained.

## Figures and Tables

**Table 1:** The chemical composition of the steel (wt. %).

Element	C	Co	Cr	Mo	Ni	Fe
mass fraction (%)	0.21	13.6	3.1	1.2	11.2	Bal

## Data Availability

Not applicable.

## References

[1] Grujicic M. (1991). Implication of elastic coherency in secondary hardening of high Co-Ni martensitic steels. J. Mater. Sci..

[2] Lee H.M., Garratt-Reed A.J., Allen S.M. (1991). Composition of M_2_C phase in tempering of high Co-Ni steels. Scr. Metall. Mater..

[3] Shi X., Zeng W., Zhao Q., Peng W., Kang C. (2016). Study on the microstructure and mechanical properties of AerMet 100 steel at the tempering temperature around 482 °C. J. Alloys Compd..

[4] Zhong P. (2007). Microstructure and Mechanical Properties in Isothermal Tempering of High Co-Ni Secondary Hardening Ultrahigh Strength Steel. J. Iron Steel Res. Int..

[5] Wang C., Zhang C., Yang Z., Su J., Weng Y. (2016). Microstructure analysis and yield strength simulation in high Co–Ni secondary hardening steel. Mater. Sci. Eng. A.

[6] Wang C.-C., Zhang C., Yang Z.-G., Su J. (2018). Carbide precipitation and element distribution in high Co–Ni secondary hardening steel. J. Iron Steel Res. Int..

[7] Wang H., Zhang J., Zhu J., Zhou F., Zhang X., Jiang Q. (2023). Structures of M_2_C carbides and its influence on strengthening in AerMet100 steel at the typical tempering temperature 482 °C. Vacuum.

[8] Lee H.M., Allen S.M., Grujicic M. (1991). Coarsening resistance of M_2_C carbides in secondary hardening steels: Part I. Theoretical model for multi-component coarsening kinetics. Metall. Trans. A.

[9] Speich G.R., Dabkowski D.S., Porter L.F. (1973). Strength and toughness of Fe-10ni alloys containing C, Cr, Mo, and Co. Met. Trans..

[10] Ayer R., Machmeier P.M. (1996). Microstructural basis for the effect of chromium on the strength and toughness of AF1410-based high performance steels. Met. Mater. Trans. A.

[11] Gruber M., Ploberger S., Wiessner M., Marsoner S., Ebner R. (2015). Influence of Heat Treatment on the Microstructure of a High Co-Ni Secondary Hardening Steel. Mater. Today Proc..

[12] Ayer R., Machmeier P. (1998). On the characteristics of M2C carbides in the peak hardening regime of AerMet 100 steel. Met. Mater. Trans. A.

[13] Ayer R., Machmeier P.M. (1993). Transmission Electron Microscopy Examination of Hardening and Toughening Phenomena in Aermet 100. Met. Trans. A.

[14] Yoo C.H., Lee H.M., Chan J.W., Morris J.W. (1996). M_2_C precipitates in isothermal tempering of high Co-Ni secondary hardening steel. Met. Mater. Trans. A.

[15] Lee H.M., Sohn H., Yoo C.H. (1997). Isothermal M_2_C carbide growth in ultrahigh strength high Co-Ni steels. Scr. Mater..

[16] Hu Z., Li X., Wu X., Wang C. (2001). HRTEM study on precipitates in high Co-Ni steel. J. Iron Steel Res. Int..

[17] Wang C., Zhang C., Yang Z., Su J., Weng Y. (2015). Analysis of fracture toughness in high Co–Ni secondary hardening steel using FEM. Mater. Sci. Eng. A.

[18] Gruber M., Ressel G., Ploberger S., Marsoner S., Kolednik O., Ebner R. (2016). Microstructural Effects on the Toughness of a High Co–Ni Steel. Adv. Eng. Mater..

[19] Gruber M., Ressel G., Martin F.M., Ploberger S., Marsoner S., Ebner R. (2016). Formation and Growth Kinetics of Reverted Austenite During Tempering of a High Co-Ni Steel. Metall. Mater. Trans. A.

[20] Wang C., Zhang C., Yang Z. (2014). Austenite layer and precipitation in high Co–Ni maraging steel. Micron.

[21] (2010). Metallic Materials—Tensile Testing—Part 1: Method of Test at Room Temperature.

[22] (2009). Standard Test Method for Linear-Elastic Plane-Strain Fracture Toughness KIC of Metallic Materials.

[23] Zhang X., Miyamoto G., Kaneshita T., Yoshida Y., Toji Y., Furuhara T. (2018). Growth mode of austenite during reversion from martensite in Fe-2Mn-1.5Si-0.3C alloy: A transition in kinetics and morphology. Acta Mater..

[24] Zhang X., Miyamoto G., Toji Y., Nambu S., Koseki T., Furuhara T. (2018). Orientation of austenite reverted from martensite in Fe-2Mn-1.5Si-0.3C alloy. Acta Mater..

[25] Zhang X.G., Miyamoto G., Toji Y., Zhang Y.J., Furuhara T. (2021). Role of cementite and retained austenite on austenite reversion from martensite and bainite in Fe-2Mn-1.5Si-0.3C alloy. Acta Mater..

[26] Wang C.-C., Zhang C., Yang Z.-G. (2017). Effects of Ni on austenite stability and fracture toughness in high Co-Ni secondary hardening steel. J. Iron Steel Res. Int..

[27] Liang Y., Long S., Xu P., Lu Y., Jiang Y., Liang Y., Yang M. (2017). The important role of martensite laths to fracture toughness for the ductile fracture controlled by the strain in EA4T axle steel. Mater. Sci. Eng. A.

